# Experiences of Australians Living With Parkinson’s Disease

**DOI:** 10.1155/padi/2661657

**Published:** 2026-02-13

**Authors:** Alycia Messing, Warren Bartik, Megan J. Hobbs, Deborah Apthorp

**Affiliations:** ^1^ School of Psychology, University of New England, Armidale, Australia, une.edu; ^2^ School of Rural Medicine, University of New England, Armidale, Australia, une.edu; ^3^ Royal Flying Doctor Service (Queensland Section), Brisbane, Australia; ^4^ School of Medicine and Psychology, Australian National University, Canberra, Australia, anu.edu.au

**Keywords:** barriers, healthcare access, patient perspectives, thematic analysis, unmet need

## Abstract

This study explores the healthcare experiences of Australians with Parkinson’s disease, focusing on healthcare access, symptom management and support networks. Despite the body of research on the experiences of PwPD, there is limited understanding of the specific challenges faced by Australians in accessing and navigating healthcare services. A national survey was conducted, and free‐text responses to an optional open‐ended question were analysed using thematic analysis to identify key themes in healthcare experiences and barriers. Seven themes were identified: Navigating Healthcare, Diagnostic Experiences, Symptom Experience and Management, Optimism and Resilience, Knowledge and Understanding of Parkinson’s Disease, Necessitated Self‐Advocacy, and Community‐Driven Support. Participants reported difficulties in obtaining timely diagnoses, navigating healthcare services and accessing specialised care. Information gaps and inadequate patient–provider communication were also noted. Peer support networks were highlighted as crucial for coping and resilience, with a notable shift away from traditional familial support structures. Findings underscore systemic challenges in healthcare access and communication for Australian PwPD and suggest that enhancing peer support networks and improving care pathways could strengthen disease management and support.

## 1. Introduction

Parkinson’s disease (PD) is a progressive neurological disorder characterised by motor and nonmotor symptoms that can significantly impact an individual’s quality of life [1]. In Australia, PD is the second most common neurological disorder, with approximately 1 in 350 individuals living with the condition [2]. Internationally, disease prevalence has nearly doubled over the past 25 years [3]. Despite treatment advancements, living with PD often requires managing a complex array of symptoms that can vary greatly between individuals [4]. As the population of people with PD (PwPD) grows, prioritising efforts to improve living conditions, access to treatment and healthcare services is critical [5].

The experiences of individuals living with chronic and degenerative disease, including PD, are highly diverse. Previous research on the lived experience of PwPD identified several unmet needs as well as barriers to care [6–8]. One of the key unmet needs faced by PwPD is the difficulty in obtaining an accurate and timely diagnosis. Delayed or incorrect diagnoses can hinder effective disease management, leading to prolonged periods of mismanagement and negatively impacting patient outcomes [9]. However, these experiences, as outlined in the literature, cannot be directly compared to the experiences of Australian PwPD, as medical and healthcare systems differ around the world. In the Australian context, healthcare is delivered through a mixed public–private system with recognised structural challenges [10]. Despite overall improvements in public perceptions of the healthcare system over the past decade, persistent concerns remain regarding adequate workforce capacity and access to care [11]. Australia’s Medicare system provides subsidised access to general practitioners (GPs) who then refer patients to specialist services, though waiting times for neurologists can be substantial, particularly in the public system. Barriers such as limited availability of specialised care, long waiting times and geographic constraints can hinder access to essential treatments and support [12–14]. For people with PD, optimal management requires coordinated multidisciplinary care involving neurologists, allied health professionals and potentially Parkinson’s specialist nurses [15]. However, the availability of such coordinated care models varies considerably across metropolitan and regional Australia, with regional areas particularly disadvantaged in access to specialist neurological services and allied health support [16]. These challenges can exacerbate the difficulties of living with PD, making it essential to examine how the current healthcare system is meeting patient needs.

In Australia, a very limited number of studies have focused on PD patients’ healthcare experiences, and these have been mostly confined to individual locations [17, 18]. To date, there are no national Australian data focusing on PD patient experience of engaging with healthcare when obtaining their diagnosis and treatment. Information gaps further complicate the management of PD. PwPD often struggle with obtaining and understanding relevant information about their condition and available treatments. Insufficient information or difficulty in navigating healthcare resources can impact medication satisfaction and result in less effective disease management and lower satisfaction with clinical encounters [19, 20]. In contrast, having more tailored information has been shown to help PwPD to feel better able to cope with their diagnosis [6]. More positive healthcare interactions are also associated with better health‐related quality of life (HRQoL) and improved physical, emotional and social outcomes [21], underscoring the importance of addressing these information gaps.

Beyond medical care, support systems play a vital role in the well‐being of PwPD. Support encompasses not only medical and therapeutic interventions but also emotional and practical assistance. Effective support involves coordinating care among healthcare providers, access to support groups and resources and ensuring that patients and their carers receive guidance and encouragement throughout their journey [5]. Support groups are particularly valuable for fostering a sense of community and independence and are often recognised by healthcare providers as a resource that alleviates the strain on overstretched healthcare systems [22]. In Australia, several state and national support bodies exist for PwPD and their carers, as do face‐to‐face and online groups, and forums. However, there is a dearth of research on their efficacy and use in the Australian context.

Despite the research exploring PD experiences internationally and within small communities, there are limited data addressing the specific unmet needs and barriers faced by Australians living with PD, including their experiences before and after diagnosis, and lived experiences. With the purpose of providing novel insights into the challenges faced by PwPD in Australia and to identify opportunities for improving care and support, this study examined the reflections of Australian PwPD on their interactions with healthcare services and their experiences of living with PD. Specifically, the following were addressed: (1) How do Australians living with PD describe their experiences of accessing healthcare and managing their symptoms? (2) What specific challenges and unmet needs do Australians with PD identify? (3) What insights do they offer for enhancing healthcare support?

## 2. Method

### 2.1. Participants

Respondents were recruited from online social media advertisements (Facebook, Instagram and Twitter) and through the Parkinson’s Australia (i.e., the peak national body for Australian PwPD) website, its state branches and social media. Posters were also placed in GP clinics, hospitals, movement clinics, physiotherapists’ and neurologists’ offices across Australia. All respondents were English‐speaking adults aged 18 years or older and self‐identified as having a diagnosis of PD.

### 2.2. Procedure

The data presented here are drawn from free‐text responses to a question from an online survey that aimed to collect predominantly quantitative data regarding PwPD’s treatment utilisation and preferences, satisfaction with current services and self‐reported HRQoL [23]. The question read ‘*Would you like to tell us anything more about your Parkinson’s symptoms or experiences accessing healthcare services? Please provide any additional comments below’*. Respondents accessed an online Qualtrics survey [version October 2021; 30] [24] through a URL or QR code from recruitment posts or posters between November 2020 and December 2021. The median completion time for the whole survey was 24 min. Given that data collection occurred during Australian COVID‐19 lockdowns, an online open‐ended survey provided a practical and low‐burden way for participants across the country to describe their experiences in their own words. Similar approaches have been shown to generate meaningful qualitative data from geographically dispersed populations and are well established in health services research [25, 26].

### 2.3. Ethics

Ethics approval for this study was obtained from the University of New England Human Research Ethics Committee (HE20‐179). Respondents provided implied consent (which included consent to be quoted anonymously) prior to commencement of the online survey.

### 2.4. Data Analysis

The data were analysed using reflexive thematic analysis as described by Braun and Clarke [27, 28] using a critical realist epistemological standpoint, acknowledging that our interpretations are influenced by both the data and our perspectives [29]. This approach was selected as it is considered robust for less rich and nuanced qualitative data, as well as for its common use in identifying patterns when answering questions related to people’s experiences, views or perceptions [28]. As the primary analyst, A.M. brought both academic and personal perspectives to the interpretive process, including disciplinary training in psychology and a background in psychological research. These lenses inevitably shaped how the data were understood and interpreted. Regular discussions with W.B., who has expertise in qualitative methods and was more distanced from the initial coding, helped to challenge assumptions and refine the thematic structure. Rather than aiming to eliminate subjectivity, we adopted a reflexive approach that recognises the active role of the researcher in theme development, consistent with the assumptions of reflexive thematic analysis [27]. The process began with familiarisation of the data through reading and re‐reading to gain an in‐depth understanding of content and context. The responses to the qualitative question were then coded, which were then grouped into provisional themes by A.M. using NVivo [30]. These themes were subsequently reviewed and refined through an iterative process among A.M. and W.B. Both the data and the themes were revisited to ensure their robustness and coherence, making necessary adjustments. Quotes reported herein have been edited to remove typographic and/or grammatical errors, with additions and changes to preserve anonymity represented by [] (brackets), and respondents are referred to by R number (e.g., R15). Each theme was defined and named to best represent its content and significance.

## 3. Results

There were 108 respondents to the full survey (87 included in a parallel quantitative study, [23]). Of these, 56 (64.4%) provided qualitative feedback on an optional open‐ended question inviting further comments about their Parkinson’s symptoms or experiences. After review, the comments of 51 (58.6%) respondents were included in this analysis. Responses excluded were those implying no comment (e.g., ‘No’, *n* = 5). Demographic and clinical characteristics of the qualitative respondents are presented in Table 1.

**TABLE 1:** Demographic and clinical characteristics of qualitative respondents (*n* = 51).

Characteristic	*n* (%)
Gender	
Female	32 (62.7%)
Male	19 (37.3%)
Age (years)	
Mean (SD)	65.77 (7.69)
Range	49–78
Relationship status	
Spouse/partner	38 (74.5%)
No spouse/partner	13 (25.5%)
Location	
Major city	22 (44.0%)
Regional/remote^∗^	28 (56.0%)
State/territory	
New South Wales	23 (45.1%)
Victoria	11 (21.6%)
Western Australia	7 (13.7%)
Queensland	6 (11.8%)
South Australia	2 (3.9%)
Tasmania	2 (3.9%)
ACT/NT	0 (0%)
Disease duration (years)	
Mean (SD)	6.75 (5.74)
Range	1–27
Disease stage (Hoehn & Yahr)	
Stage 1 (unilateral symptoms only)	20 (39.2%)
Stage 2 (bilateral symptoms, no balance impairment)	7 (13.7%)
Stage 3 (bilateral symptoms, balance impairment)	14 (27.5%)
Stage 4 (severe disability, still able to walk)	7 (13.7%)
Stage 5 (wheelchair bound or bedridden)	0 (0%)
Unsure	3 (5.9%)
Healthcare service utilisation	
Currently seeing a GP	37 (72.5%)
Currently seeing a neurologist	39 (76.5%)
Currently accessing allied health services^∗∗^	27 (52.9%)
Member of PD support group	21 (41.2%)

^∗^Regional/remote includes inner regional, outer regional, remote and very remote areas as classified by ASGS 2021–2026.

^∗∗^Allied health services may include physiotherapy, occupational therapy, speech therapy, dietetics, exercise physiology, psychology or other allied health professionals.

**FIGURE 1:**
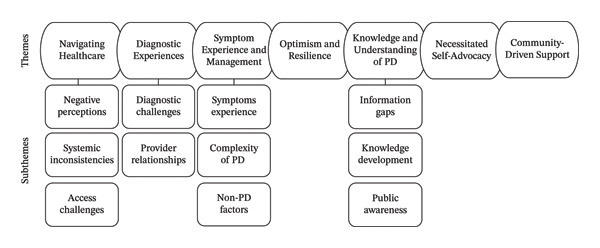
Thematic map of the themes and subthemes of the lived experiences of PwPD.

Seven major themes and 11 subthemes were identified. As can be seen in Figure 1, these included (1) Navigating Healthcare, (2) Diagnostic Experiences, (3) Symptom Experience and Management, (4) Optimism and Resilience, (5) Knowledge and Understanding of Parkinson’s Disease, (6) Necessitated Self‐Advocacy and (7) Community‐Driven Support.

### 3.1. Theme 1—Navigating Healthcare

#### 3.1.1. Negative Perceptions

Respondents expressed negative attitudes towards the current healthcare system, citing frustrations with various aspects, including the system’s overall efficiency, specific components of care, the processes and the accessibility of services. One respondent (R4), with extensive years of experience, remarked

*‘Living with Parkinson’s for more than 23 years has given me unique insight into the provision of healthcare services to the xxx Australians currently living with this complex and challenging illness. Parkinson’s patients need a coordinated multidisciplinary model of care provided by a team of health professionals with specialist training in the management of Parkinson’s. It is incredibly frustrating that in 2021, Parkinson’s related healthcare services are ad hoc, overly focused on neurology services and dependent on luck over design for the establishment and success of many programs’.*

#### 3.1.2. Systemic Inconsistencies

While many recognised the need for symptom management to involve interdisciplinary collaboration, they also recognised inconsistencies in the healthcare system. Respondents noted variability in clinician expertise, availability of services and coordination between providers. As one respondent (R43) commented, the ‘*quality of neurologists is variable—from very good to poor’.*

#### 3.1.3. Access Challenges

Access issues were also highlighted, with one respondent (R19) describing the difficulty obtaining continuous care:

*‘In the town that I live, we have no permanent doctor, they are all Locums. It makes it extremely hard to get continuous care. I live an hour away from any bigger towns but travel for me is extremely hard’.*

Others discussed the challenge of accessing neurologists, citing long distances and extended travel times, as illustrated by one respondent’s (R17) experience, ‘*have to do own research to find out what or who is available. Been trying to get access to local neurologist, 200 kms away for over 12 months’*.

### 3.2. Theme 2—Diagnostic Experiences

#### 3.2.1. Diagnostic Challenges

Respondents shared a range of experiences and interactions with healthcare professionals regarding their PD diagnosis and management. This included positive experiences, for instance, when their diagnosis provided clarity:

*‘My diagnosis of PD was an answer to the many symptoms I had been experiencing. I thought I had MS because I had little knowledge of PD and thought only “old” people had it. My symptoms improved dramatically with Medication. I see my neurologist as a private patient and have not used the public system’.* (R15)

Negative experiences were marked by scepticism or resistance, as demonstrated by a respondent (R29) who reported that *‘my GP originally thought I was a hypochondriac sent me to a psychologist. Believed my symptoms were in my head and I was making them up’*. Another respondent (R45) also recounted the difficulties in obtaining a diagnosis:

*‘I had a round trip of 960k to get a diagnosis of Parkinson’s from a Neurologist as locally was a 12 to 18 month wait for appointments. I had changed GP’s twice trying to get a diagnosis with no luck, eventually my physiotherapist suggested I Google my symptoms (he knew but wasn’t qualified to say). I then had to demand a referral from my GP and get an appointment with a neurologist in [capital city] myself. Turns out I have a brother and uncle with Parkinson’s and it’s not supposed to be genetic’.*

Some respondents also described clinicians expressing surprise at their diagnosis, reflecting the subtlety of early symptoms. One respondent (R34) noted that their GP was ‘very surprised’ by the diagnosis despite prodromal symptoms and a family history of PD:

*‘I asked my GP for a referral to a Neurologist as I had noticed subtle changes in myself—lack of facial expression, deterioration of my handwriting, constipation, raspy voice, fatigue. I was aware of these symptoms as my mother was diagnosed with Parkinson’s at 85. My GP was very surprised at my diagnosis. I am fortunate to have mild Parkinson symptoms with little progression in 9 years. I have done my own research to find services appropriate for me’.*

#### 3.2.2. Provider Relationships

Some respondents reflected on their interactions with medical practitioners and felt that they were not being listened to when expressing their symptoms or their needs ‘*accessing health care services is difficult. The neurologist does not listen to my symptoms’* (R22).

### 3.3. Theme 3—Symptom Experience and Management

#### 3.3.1. Symptoms Experience

Many respondents shared their symptoms and provided feedback on how they perceived their symptoms had changed with treatment. One respondent (R46) reflected that ‘*my main symptoms involve facial tremors and dental issues, and a gradual increase in balance’*. Others described factors that are known to impact HRQoL ‘*worst thing is sleeping or lack of it’* (R44).

Commentary on how respondents experienced their symptoms changing with treatment was also common. Many shared positive sentiments about their medications:

*‘I never lost my sense of smell. I respond very well to [medication] no side effects yet … but if I miss a dose and I get very slow and heavy. I have regular dry eye and excess saliva. Exercise is so important but I lack motivation. I just have had my drivers license restricted which could have been avoided if the competency test wasn’t so expensive $700’.* (R11)

Others reported that physical exercise was central to their treatment program and symptom management, with one respondent (R32) reported that

*‘Having access to services that promote exercise has also been a critical factor in keeping my symptoms manageable. I attend Tai Chi once a week, 2 dance/ exercise classes a week, monthly exercise class with Occupational Therapist (when not in lockdown) as well as 1 hour 30 minutes of mixed exercise/stretches daily at home’.*

#### 3.3.2. Complexity of PD

When reflecting on their symptom experience, some respondents acknowledged the complexity of PD, as illustrated by this respondent (R35) ‘*I had no idea about how wide the range of symptoms is, or how utterly exhausting it is. Dragging a reluctant non-complying body around is undignified, ugly and just plain tiring’.*

#### 3.3.3. Non‐PD Factors

Others highlighted that PwPD are more than their diagnosis and can be susceptible to other health problems that may be overshadowed by their condition:

*‘The number one frustration for me with having a* diagnosis *of [young onset] YOPD is that when presenting to the GP - symptoms are first looked at as being part of the diagnosis rather than additional. We are not immune to everything else’.* (R1)

### 3.4. Theme 4—Optimism and Resilience

Not all comments about the healthcare system and PD were negative. Some respondents reported a more optimistic view. These individuals perceived PD as a more manageable disease and reported more positive sentiments towards their diagnosis, outcomes and overall experience with the disease. One respondent (R27) approached their condition with a light‐hearted attitude, stating ‘*life is good, except when I spill my beer’.* Another respondent (R37) reflected on how their diagnosis became a catalyst for positive lifestyle change: ‘*My diagnosis was the beginning of a more fulfilling lifestyle’.*

Positive perspectives also extended to healthcare access: ‘*provision of health care services is excellent’* (R40). Others expressed their appreciation for the professionals they had interacted with, as illustrated by this respondent (R49): ‘*[hospital] have the most amazing group of people in the Parkinson Clinic. I could not ask for better help’*. Additionally, one respondent (R13) reflected on the positive experiences they had had with support groups and their ability to access information:

*‘I was living in [capital city] when diagnosed, with a good working knowledge of how health services work, I have been able to access good services in [city] since I moved here. There are also good resources online, including webinars through Parkinsons [state]. The local community health service runs a PD [program] which is very helpful. I wonder how people cope if less service literate, relying on GPs to diagnose/refer, particularly those who are young and active’.*

### 3.5. Theme 5—Knowledge and Understanding of PD

#### 3.5.1. Information Gaps

Many respondents commented on their experiences with information availability, highlighting issues such as the lack of information provided during consultations about treatment, PD, healthcare services and government support. One respondent (R50) commented

*‘I feel that both my GP and specialist tend to assume that the internet will provide all the information I need or want in regard to this disease. I’m fortunate that I can access and understand what I need to know but a lot more input from the medical profession would be appropriate’.*

Other respondents noted the lack of information about alternative or government support services, for example,

*‘I was diagnosed on a Tuesday on Thursday seeked* assistance *by myself. Neurologist did not inform me of any services. Not enough information of health care services. Definitely not enough information of government services. E.g. SWEP [State-wide Equipment Program] or My Aged Care’.* (R28)

One respondent (R30) experienced information delays and only gaining information about available resources through their connections with support communities, leading to frustrations over missed opportunities:

*‘I was totally unaware of access to the NDIS [National Disability Insurance Scheme] until informed by a visiting Parkinson’s [state] guest speaker to our support group which I run until it was too late. I had turned 65 three weeks before her visit! Why are people not informed of these things? How are they meant to find out?’*

#### 3.5.2. Knowledge Development

In addition to concerns about information availability, several respondents expressed frustration over the limited recognition of nontraditional symptoms of PD. These symptoms, though experienced by many, are often overlooked or inadequately addressed during medical consultations and in available resources. As one respondent (R12) noted: ‘*lots of things that aren’t listed as symptoms are experienced by a great number of people who have Parkinson’s. They’re never listed and could provide more insight into this disease’*.

#### 3.5.3. Public Awareness

Beyond personal experiences with information availability, some respondents highlighted the lack of sufficient education about PD for the general population. Concerns were expressed that while some progress has been made, there is still a significant need for better education and resources. One respondent (R26) reflected that

*‘It’s quite a bit different now, than it was in 2013 when I was first diagnosed, so I can’t be sure what information is given to people. In 2013 it was woeful. Cost is a big factor. I have been fortunate enough to be deemed eligible for an NDIS package which is paying for my PD [program] group classes and 8 weekly sessions with an exercise physiologist. With PD being said to be reaching epidemic proportions there is a massive need to educate about PD’.*

### 3.6. Theme 6—Necessitated Self‐Advocacy

For many respondents, their healthcare experiences have led to them having to take responsibility for coordinating their own care, seeking information independently and making decisions in the absence of sufficient guidance from health professionals. For some individuals, this has also meant educating healthcare professionals; one respondent (R35) expressed that ‘*there is little information supplied about available services, it is sheer luck to find anything relevant. A practice nurse told me she also had little provided to her, she learnt mostly from PD patients’.*

Due to a lack of readily available information and support, many respondents found themselves independently seeking out the resources they needed. As one respondent (R2) put it: ‘*I had to go hunting for the supports to receive. It should be easier’*. Others leveraged their previous professional experience or expertise to navigate the healthcare system effectively. For example, one respondent (R8) noted

*‘I worked in Neuro rehab for 15 years at time of diagnosis, I was lucky to be able to see neurologist and seek treatment without waiting. Most people slip through the cracks and don’t get the advice and support they require to live their best life with PD’.*

### 3.7. Theme 7—Community‐Driven Support

Respondents described turning to community‐driven forms of support, including online groups and informal networks, which provided practical advice and shared experiences and a sense of solidarity that compensated for the limited availability of formal resources. Many respondents expressed a heightened sensitivity to the challenges faced by fellow individuals with PD, reporting a sense of empathy and concern:

*‘There is a shortage of neurologists in the [region]. Currently 2000 new patients waiting to see a neurologist, and 1-2 years wait. As an individual this is ok but as leader of a support group I am dismayed at the problems this is causing for other people living with Parkinsons in my area’.* (R36)

This awareness often highlighted a contrast between their own experiences and those of others, leading to feelings of being ‘lucky’, and a sense of gratitude. One respondent (R24) noted that there is ‘*no access to Parkinson’s nurse in the regional areas of [state]. I’m ok for now … but many others are not. More funding required for support to implement services needed in regional areas’.*

Respondents frequently conveyed appreciation for the support and connections within the PD community. The value of being part of a network that provides mutual aid and understanding, which can significantly impact their overall experience with the disease, was recognised, including by one respondent (R7):

*‘Social media and connections via Facebook for PLWP have been a godsend. Even TikTok has hash-tagged PLWP, which helps you feel you are not alone. The original GP who suggested I had PD, was particularly clinical and heartless in his approach, there should be counselling recommended on how to handle this diagnosis’.*

## 4. Discussion

To date, little attention has been devoted to understanding the lived experience of PwPD in the Australian context. This study posed three research questions: How do Australians living with PD describe their experiences of accessing healthcare and managing their symptoms, what specific challenges and unmet needs do Australians with PD identify and what insights do they offer for enhancing healthcare support? The aim of the study was to help understand PwPD’s lived experience and address gaps in knowledge by analysing free‐text comments from national survey. This is one of the few studies to obtain responses from PwPD on a national level in Australia. Thematic analysis identified seven themes: Navigating Healthcare, Diagnostic Experiences, Symptom Experience and Management, Optimism and Resilience, Knowledge and Understanding of Parkinson’s Disease, Necessitated Self‐Advocacy and Community‐Driven Support. These themes provide insight into the personal experiences of PwPD, focused on symptoms and experiences of healthcare and services access and highlight both challenges and opportunities for improving the experience of living with PD.

A recurrent theme among respondents was frustration with the inefficiencies and variability of healthcare services, particularly in terms of access to specialised care. Consistent with prior research [8, 13, 31], many respondents noted long waiting times and the difficulty of obtaining continuous care, especially in regional areas. This mirrors findings in rural healthcare, where geographical isolation often leads to delayed diagnoses and limited specialist availability [32]. As highlighted by one respondent, waiting up to 12 months for an appointment with a neurologist is not uncommon, a concern that aligns with the broader literature on healthcare disparities in rural Australia [12]. These access challenges are particularly problematic in managing a progressive disease such as PD, where timely interventions can significantly influence the quality of life [33]. The interdisciplinary care model is recognised as the best practice for managing PD [32] and is present in Australia; however, its effectiveness is often limited, particularly in settings that lack a multidisciplinary approach, leading many PwPD to rely primarily on neurology services without consistent access to allied health professionals [34]. This lack of coordination may decrease patients’ overall satisfaction with their services.

Communication between healthcare providers and patients was often reported as suboptimal. Many respondents felt that their concerns were not taken seriously or that they were not provided with sufficient information about their diagnosis or treatment options. These experiences reflect gaps in PD‐specific training, particularly in recognising nonmotor and early‐stage symptoms [35, 36]. When GPs lack familiarity with the subtle and varied presentation of PD, patients’ concerns may be minimised or dismissed entirely, contributing to diagnostic delays and necessitating patient self‐advocacy [37, 38]. The fragmented nature of care delivery, combined with time constraints in standard consultations, further compounds these communication barriers. This reflects broader concerns in the literature regarding the quality of patient–provider interactions and the need for more patient‐centred care [7, 39]. Improving diagnostic communication and providing more comprehensive information during the early stages of the disease may help alleviate patient frustrations and improve long‐term outcomes [8].

The respondents also reflected on their difficulty obtaining relevant information about their condition and available treatments. This aligns with previous research indicating that information gaps can lead to lower satisfaction with healthcare services and poorer disease management outcomes [6, 20]. Many respondents indicated that they had to rely on self‐research or support groups to obtain critical information about their disease, suggesting that healthcare providers may not be sufficiently addressing these informational needs. Tailored information and guidance, particularly regarding government support services, were also identified as areas of concern. Several respondents only became aware of these services through support groups or after it was too late to apply, further underscoring the need for improved communication and education at the point of diagnosis.

Despite these challenges, some respondents reflected on the positive aspects of their healthcare experience and the importance of support systems in managing their condition. Access to community‐based exercise programs, support groups and tailored therapies was highlighted as beneficial for maintaining physical function and that may contribute to overall quality of life. These findings support the existing literature on the importance of nonpharmacological interventions in PD management [40].

An interesting observation from these data was the absence of any mention of partners in any comments, despite 74.5% of respondents reporting having a spouse or partner (see Table 1). This contrasts with much of the existing literature, where partners are typically seen as key sources of emotional and practical support and frequently mentioned [7, 41]. However, this absence should be interpreted cautiously, as the question itself focused on healthcare services and symptom experiences rather than explicitly prompting reflection on support systems or relationships. The lack of partner mentions may reflect the specific framing and scope of the question, the limitations of single open‐ended question data collection or potentially evolving patterns in how some Australians with PD conceptualise their primary support networks, with greater emphasis on peer‐based and community‐driven support as evidenced in Theme 7. This preliminary observation warrants further investigation through more targeted inquiry methods, such as semistructured interviews, to better understand the role of partners in the Australian PD context and how this might differ from other international contexts.

Community‐driven support structures emerged as significant, particularly the role of peer‐based networks. Although participants did not reference formalised support models, the emphasis on shared lived experience and practical advice points to the value of informal peer connections. These networks, often sustained through local groups or online platforms, can serve as critical emotional and informational lifelines for people with Parkinson’s [22, 42]. The lack of partner mentions, alongside the emphasis on peer‐based support, may reflect evolving patterns in how some individuals seek and receive care in the Australian PD context, although further investigation is needed to determine whether this represents a broader shift. This underscores a practical implication: the need for a more fostering and support environment of these networks. The benefit PD nurses have on PwPD has been well documented [43]. Enhancing these networks could provide more reliable and accessible emotional, practical support and reduce costs [44, 45].

Much of the research around PD tends to focus on the challenges and limitations associated with the disease [15]. However, the experiences shared by respondents in this study offer an opportunity to change this perspective, focussing on resilience, peer support and personal empowerment. By improving the support offered through peer networks and fostering more comprehensive patient networks, there is potential to reshape the lived experience of PD. Such a shift could help individuals with PD reframe their experience, reducing the sense of isolation and helplessness that often accompanies the disease [46]. Such networks could represent a resource that could be more actively cultivated to improve factors contributing to the quality of life for PwPD.

The limitations of this study should be acknowledged. One major limitation was the underrepresentation of younger individuals with PD and the absence of participants with later‐stage PD, which limits the generalisability of the findings across the broader PD population. The question posed was singular and framed around services and symptoms, which limited the depth and breadth of participant responses. The use of a single online open‐ended question, while enabling broad national reach, restricted opportunities for follow‐up, clarification of ambiguous responses or probing of emerging themes. This format likely influenced the richness of the qualitative data obtained and may have narrowed the scope of responses, potentially contributing to observations such as the absence of partner mentions despite a high proportion of partnered individuals in the sample. Respondents with more complex experiences or difficulty articulating thoughts in writing may have been less likely to provide detailed responses, introducing the possibility of bias towards those more comfortable with written expression. Although this method is recognised as a practical approach for collecting dispersed qualitative perspectives, particularly during pandemic conditions, it cannot achieve the depth offered by semistructured interviews or multistage qualitative designs. Future research would benefit from targeting a broader age and severity range and incorporating methods such as face‐to‐face or online interviews, or offering follow‐up opportunities, to enable the collection of richer, more detailed data. This highlights the importance of listening more closely to the voices of PwPD and recognising the value of their lived experience.

Despite these limitations, this study boasts several notable strengths. The Australia‐wide reach, including a near‐equal representation from regional and metropolitan areas, provides valuable insights into place‐based differences in the lived experiences of PwPD. While the range of disease durations could be seen as a limitation due to the lack of a focus on stages, it also adds breadth to the dataset. This diversity offers perspectives from individuals at various points in their Parkinson’s journey, which enhances the ecological validity of the findings. While the analysis did not formally compare coping strategies by disease stage, the inclusion of this range allows for a more nuanced understanding of how individuals navigate the condition across varying contexts.

## 5. Conclusions

This study contributes important insights into the lived experiences of Australians with PD, highlighting gaps in service accessibility, communication and support, particularly within regional and rural contexts. The findings reinforce concerns raised in prior research regarding the fragmented nature of healthcare delivery but also draw attention to underexplored aspects of the Australian context, such as the limited visibility of partner involvement in coping narratives. The prominence of peer‐based support networks, both in‐person and online, suggests a potentially underutilised avenue for bolstering emotional and practical support among PwPD. These informal systems may play a critical role in filling gaps left by formal services, especially where access is restricted or inconsistent. Enhancing and legitimising such networks within models of care could foster greater patient empowerment and mitigate the isolation that often accompanies chronic illness. The themes of resilience, self‐advocacy and positivity evident across responses offer a counter‐narrative to deficit‐based framings of PD. By foregrounding these experiences, the findings support a broader shift towards strength‐based approaches that recognise the agency and adaptability of PwPD. Future research should further examine the nuanced social dynamics of care, including the role of partners, and explore strategies to integrate patient voice more effectively into service design. Doing so may not only improve HRQoL but also contribute to a more responsive and person‐centred model of care for those living with PD in Australia.

## Author Contributions

Alycia Messing contributed to the study conception and design, material preparation, data collection, conceptualised analysis, performed analysis and wrote and reviewed the manuscript. Warren Bartik contributed to the data analysis, data visualisation and manuscript review. Megan J. Hobbs and Deborah Apthorp contributed to the design, materials and manuscript review.

## Funding

This research was supported by an Australian Government Research Training Program (RTP) Scholarship and was not industry sponsored.

## Ethics Statement

Ethics approval for this study was obtained from the University of New England Human Research Ethics Committee (HE20‐179). Respondents provided implied consent (which included consent to be quoted anonymously) prior to commencement of the online survey.

## Conflicts of Interest

The authors declare no conflicts of interest.

## Data Availability

The data that support the findings of this paper are available from the corresponding author upon reasonable request.
